# Genomic Characterization of Interspecific Hybrids between the Scallops *Argopecten purpuratus* and *A. irradians irradians*

**DOI:** 10.1371/journal.pone.0062432

**Published:** 2013-04-19

**Authors:** Liping Hu, Xiaoting Huang, Junxia Mao, Chunde Wang, Zhenmin Bao

**Affiliations:** 1 Key Laboratory of Marine Genetics and Breeding (MGB), Ministry of Education, College of Marine Life Sciences, Ocean University of China, Qingdao, China; 2 College of Marine Science and Engineering, Qingdao Agricultural University, Qingdao, China; Auburn University, United States of America

## Abstract

The Peruvian scallop (*Argopecten purpuratus*) has been introduced to China and has successfully been hybridized with the bay scallop (*A. irradians irradians*). The F1 hybrids of these two scallops exhibited a large increase in production traits and some other interesting new characteristics. To understand the genetic basis of this heterosis, nuclear gene and partial mtDNA sequences, and genomic *in situ* hybridization (GISH) were employed to analyze the genomic organization of the hybrids. Amplification of the ribosomal DNA internal transcribed spacer (ITS) showed that the parental ITS sequences were present in all the hybrid individuals, illustrating that the hybrid offspring inherited nuclear DNA from both parents. Sequence analyses of the ITS region further confirmed that the hybrids harbored alleles from their parents; some recombinant variants were also detected, which revealed some alterations in the nuclear genetic material of the hybrids. The analysis of mitochondrial 16S rDNA showed that the hybrids possessed sequences that were identical to the 16S rDNA of the female parents, proving a matrilineal inheritance of mitochondrial genes in scallops. In addition, GISH clearly discriminated between the parental chromosomes and indicated a combination of haploid genomes of duplex parents in the hybrids. The genetic analyses in our study illustrated that the F1 hybrids inherited nuclear material from both parents and cytoplasmic genetic material maternally, and some variations occurred in the genome, which might contribute to a further understanding of crossbreeding and heterosis in scallop species.

## Introduction

Distant hybridization is not only a useful way of producing heterosis but also an effective method of expanding the source of species variation and breeding new varieties. Interspecific crosses between divergent species have been widely performed in aquaculture to induce hybrid vigor [1], [2], and some studies have shown that distant hybrids can present a wide range of genetic variation, including the hybrids of *Clarias macrocephalus* × *Pangasius sutchi* [3], *Ctenopharyngodon idella* × *Cyprinus carpio* [4], [5] and *Paralichthys olivaceus* × *P. dentatus* [6]. Such variation has provided the conditions for genetic improvement in the aquaculture industry.

Scallops are one of the most ecologically and economically important bivalve groups in the world. Crossbreeding among species has been attempted to improve the productivity and disease resistance of some commercial scallop species, and these efforts have proven the feasibility of this approach [7]–[12]. The research regarding the genomic constitution and variation of interspecific hybrids has indicated some interesting genetic phenomena, such as the report of Lü et al. [10] that some hybrid progeny of *Chlamys farreri* ♀ × *Patinopecten yessoensis ♂* were haploid, triploid and aneuploid, non-equivalent chromosome inheritance and some gynogenesis-like individuals. The analysis of the chromosomal components in hybrids between *Mimachlamys nobilis* and *C. farreri* by Huang et al. [12] indicated that most hybrids contained 35 chromosomes, corresponding to the theoretical expectation of hybrids between these two species, but a few gynogenetic individuals, chromosome fragmentations, aneuploids and allopolyploids were also detected in some F1 individuals. The above analyses of the genomic constitution of interspecific hybrids and the previous studies about heterosis in scallops have mostly focused on hybrid embryos at an early developmental stage, mainly because viable adult hybrid scallops have been rarely reported. Although Yang et al. [13] reported that adult hybrids were obtained from interspecific crosses of *C. farreri* × *P. yessoensis*, the investigation of the genetic composition of the adult hybrids indicated that no true hybrid could be confirmed [9].

The bay scallop *Argopecten irradians irradians* (Lamarck, 1819) is naturally distributed along the Atlantic coast from New Jersey northward to Cape Cod [14]. This species was introduced to China from the US in 1982 [15], and a vast aquaculture industry based on the scallop soon developed in northern China. The Peruvian scallop *A. purpuratus* (Lamarck, 1819) is naturally distributed along the Pacific coast of South America [16]. These two scallops are both hermaphroditic *Argopecten* scallops and have the same chromosome number, with similar karyotypes [17], [18]. However, the animals have distinct sizes, life spans and temperature tolerance [19]. In 2007 and 2008, the Peruvian scallop was introduced to China and was successfully hybridized with the bay scallop. The hybrid offspring exhibited extraordinary heterosis in growth in addition to some new features [19]. However, the genomic constitution of the hybrids and the contribution rate of the parents to the hybrids have remained unclear.

To understand the genetic basis of the observed heterosis, we analyzed the genome organization of the *A. i. irradians* × *A. purpuratus* hybrid offspring. The inheritance of nuclear and mitochondrial genetic material from the parents to the F1 progeny was first investigated through an analysis of the nuclear gene and mtDNA sequences, involving the internal transcribed spacer (ITS) region and 16S rDNA, respectively. Genomic *in situ* hybridization (GISH) was then employed to identify the chromosomal composition of the hybrid larvae at the cytogenetic level.

## Results

The ITS region was successfully amplified in all of the 40 individuals tested, and the results of polyacrylamide gel electrophoresis are shown in Fig. S1. A band of approximately 770 bp was amplified using Peruvian scallop DNA, and a set of larger bands (780 bp) were amplified for the bay scallops. Both types of bands were amplified using DNA from the hybrids (PB and BP), indicating that the hybrids inherited nuclear DNA from both parents. The presence of multiple bands for some parents and hybrid individuals, such as in Fig. S1, lines 3, 4, 7, 10 and 11, could be attributed to the ITS diversity among different repeat units.

To further investigate the ITS sequence information in the genome of the hybrids, the amplified ITS products from the parents (PP and BB) and hybrid offspring (PB and BP) were cloned and sequenced. The total length of the ITS region was 773–785 bp, with 46.6–47.3% GC content, in PP and 767–771 bp, with 47.0%–47.6% GC content, in BB. The alignment of the ITS sequences showed 771 identical pairs, 3 transitional pairs and 4 transversional pairs in PP and 767 identical pairs, 2 transitional pairs and no transversional pairs in BB. The homology of the ITS regions between PP and BB was estimated at 96.3% and 99.5% (PP) and 99.8% (BB) intraspecies. The ITS sequences homologous to PP and BB were both present in the hybrids.

**Figure 1:**
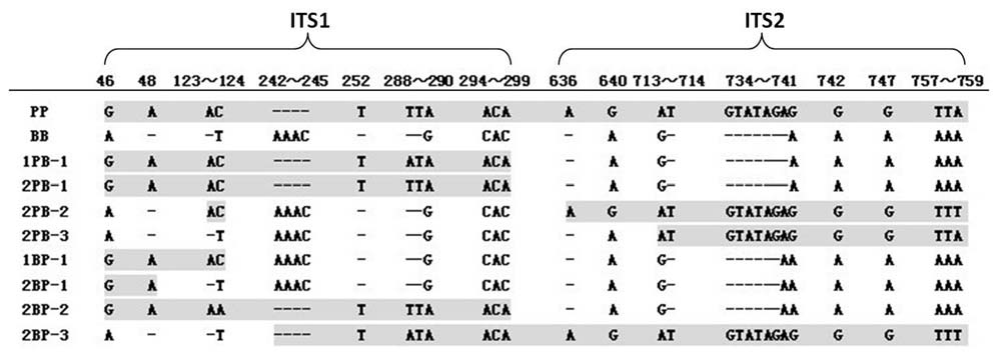
Polymorphic sites in the ITS sequences of the parental representations and 8 recombinant variants. PP, *A. purpuratus*; BB, *A. i. irradians*; mPB-n and mBP-n, recombinant variants (PB, *A. purpuratus* ♀ × *A. i. irradians* ♂, and BP, *A. i. irradians* ♀ × *A. purpuratus* ♂), whereby m is the individual number and n is the clone number. The sequences homologous to those of *A. purpuratus* are shown with a black background. (ITS1, 34–314 bp; 5.8S, 315–471 bp; ITS2, 472–761 bp).

An alignment analysis of all the ITS sequences of the parents and hybrid offspring showed that the polymorphism was caused by indels and nucleotide substitutions, with all indel polymorphic sites being located in the central regions of ITS1 and ITS2. In 96 randomly selected clones from the hybrid individuals, 88 were identified as representative of parental ITS alleles, involving half of the PP alleles and half of the BB alleles. The number of these two types of alleles in each hybrid individual was also nearly equal. In addition, eight recombinant variants in four individuals were identified and comprised segmental sequences of PP and BB. A simplified alignment of the parental ITS representatives and eight recombinant variants are shown in Fig. 1. ITS1 region was from 34 bp to 314 bp, and ITS2 region was from 472 bp to 761 bp. 5.8SrDNA was the sequences between ITS1 and ITS2 (315–471 bp), which was highly conserved. Of the eight recombinant variants, the first part of the amplified ITS region of 1PB-1, 2PB-1, 1BP-1, 2BP-1 and 2BP-2 contained sequences from PP, whereas the second part of these amplified ITS region contained sequences from BB. In contrast, the first part of the amplified ITS region of 2PB-3 and 2BP-3 contained sequences from BB, and the second part contained sequences from PP. For 2PB-2, the first and the third parts of the amplified ITS region contained sequences from BB, whereas the second and last parts of the amplified ITS region contained sequences from PP.

**Figure 2:**
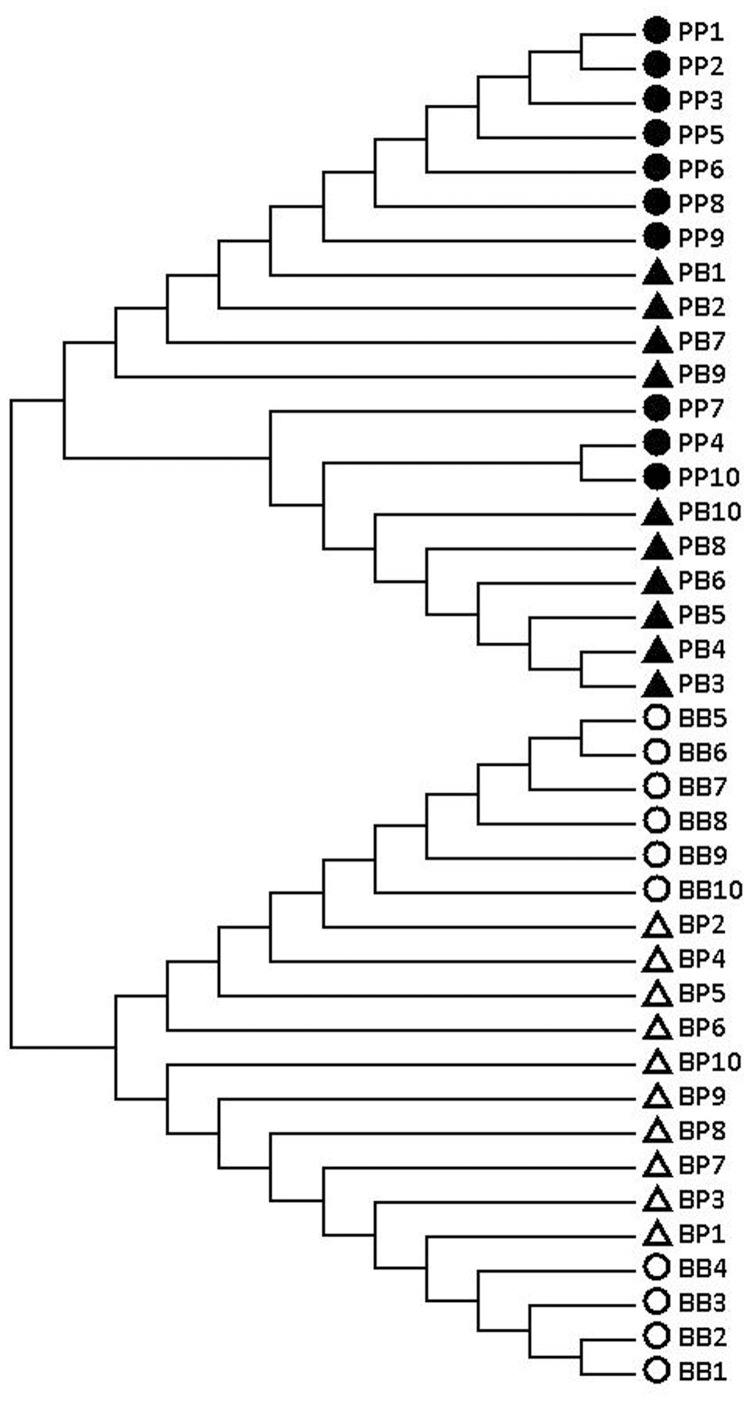
The Bayesian tree for *A. purpuratus* (PP•), *A. i. irradians* (BB○) and their hybrids of *A. purpuratus* ♀ × *A. i. irradians* ♂ (PB▴) and *A. i. irradians* ♀× *A. purpuratus* ♂ (BP△), as inferred from mitochondrial 16S rDNA sequences.

A total of 542 nucleotides of the 16S rDNA fragment from PP, BB, PB and BP were successfully amplified and sequenced. After alignment, 480 conserved sites, 62 variable sites, 44 parsimony-informative sites and 18 singleton sites were found, of which the variable sites were all transitions or transversions; in contrast, insertions/deletions were not found. The homology of the 16S rDNA fragment between BB and PP was 95.8% and 99.5% (BB) and 99.6% (PP) intraspecies. The 16S rDNA sequences of BP had a high homology with the female parent BB (99.6%), and the homology of the 16S rDNA between PB and PP was also 99.6%, which was equivalent to that within the BB or PP species. The data for the genetic distance between groups were consistent with the results of the homology analysis. The phylogenetic analysis based on the Bayesian method clearly divides the individuals into two clusters (Fig. 2): one cluster includes all the BB and BP individuals, and the other cluster is formed by all the PP and PB individuals. In short, the hybrids possessed 16S rDNA sequences that were almost identical to their female parents. Thus, 16S rDNA could be utilized as a useful marker for the distinction of reciprocal-cross hybrids.

**Figure 3:**
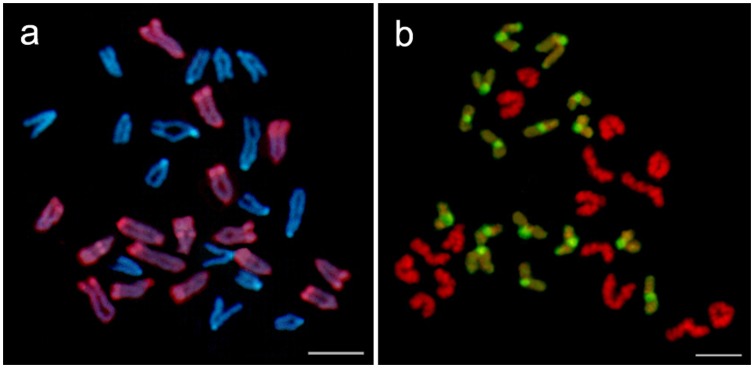
Representative metaphase chromosomes of the F1 hybrids of *A. purpuratus* × *A. i. irradians* examined by GISH. In (a), the total genomic DNA from *A. i. irradians* is used as the probe. The chromosomes are stained with rhodamine (red) and counterstained with DAPI (blue). In (b), the total genomic DNA from *A. purpuratus* is used as the probe. The chromosomes are stained with FITC (green) and counterstained with PI (red). Bars = 5 µm.

**Figure 4:**
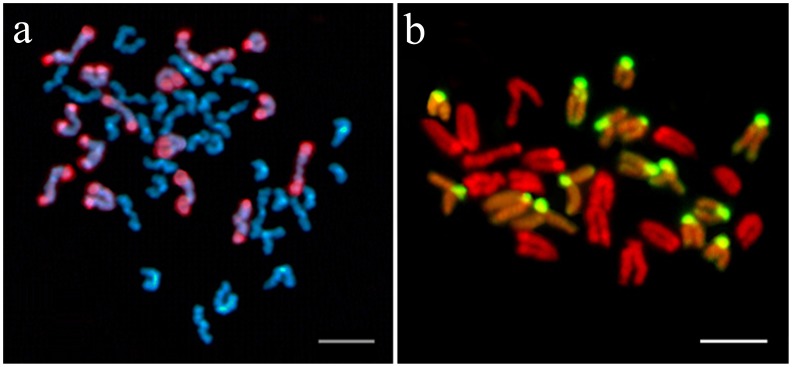
Examples of allotriploid (a) and aneuploid (b) in the F1 hybrids examined by GISH. (a) Chromosomes are identified using the *A. i. irradians* genomic DNA probes. The 16 (n) chromosomes originating from *A. i. irradians* are shown with rhodamine (red), and the other 32 (2 n) chromosomes originating from *A. purpuratus* are counterstained with DAPI (blue). (b) Chromosomes are identified using the *A. purpuratus* genomic DNA probes. The green chromosomes are from *A. purpuratus*, and the red ones are from *A. i. irradians*. One chromosome from *A. purpuratus* and one chromosome from *A. i. irradians* were eliminated in the metaphase spread. Bars = 5 µm.

The GISH technique effectively distinguished all of the chromosomes inherited from *A. i. irradians* and *A. purpuratus* in the hybrids, and it revealed that the haploid chromosomes from both parents were intact in most of the hybrids (73.7% in PB and 66.7% in BP). Fig. 3 (a) shows that, when using digoxigenin-labeled genomic DNA probes from *A. i. irradians*, 16 chromosomes originating from *A. i. irradians* hybridized with the probes and were colored red; in contrast, the other 16 chromosomes from *A. purpuratus* were counterstained with DAPI and were colored blue. Similarly, in Fig. 3 (b), the chromosomes in the hybrids originating from *A. purpuratus* were stained green by biotin-labeled genomic DNA probes from *A. purpuratus*, whereas the chromosomes from *A. i. irradians* were counterstained with PI and colored red. Additionally, about 30% aneuploids and allopolyploids were observed in some F1 hybrids, as shown in Fig. 4. Moreover, GISH showed that the fluorescent signals were not uniform among the chromosomes, with the stronger signals showing banding patterns on different chromosomes (termed GISH banding). As shown in Fig. 3, stronger hybridization signals were found mainly in the peri-centromeric and/or centromeric regions of all the chromosomes from *A. purpuratus*. Conversely, stronger signals were mainly distributed on the telomeric and/or peri-telomeric regions of all the chromosomes and the interstitial regions on the long arm of some chromosomes of *A. i. irradians*.

## Discussion

The feasibility of crossbreeding has been tested among many commercial scallop species, such as *C. farreri*, *A. i. irradians*, *P. yessoensis* and *M. nobilis*, and the genomic organization and variation of the hybrids have also been described [7]–[12]. In these experiments, the hybrid larvae were found to be viable, whereas viable adult hybrids were not found. Yang et al. [13] reported that adult hybrids from *C. farreri* × *P. yessoensis* were obtained; however, the identity of hybrids could not be confirmed by GISH and RAPD analysis, and no paternal genetic material was detected in the hybrid adults [9]. In contrast, *A. purpuratus* × *A. i. irradians* hybrid larvae were verified by GISH in the present study, and the hybrid adults were confirmed to be true hybrids by the ITS analysis. The evidence based on GISH analysis provided convincing support at the cytogenetic level for the conclusion that the hybrid offspring of *A. purpuratus* × *A. i. irradians* inherited their nuclear genomic material from both parents and that they were true hybrids. Moreover, the amplification with ITS primers using hybrids DNA produced both parental bands, revealing that both parents had contributed to the genome composition of the hybrids. However, it should be noted that the lengths of the sequenced PP and BB ITS regions were inconsistent with the electrophoresis results, which might be attributed to different molecular configurations of the amplified ITS fragments from PP and BB during polyacrylamide gel electrophoresis. Regardless, there is no doubt that the ITS region can play an important role in hybrid identification. The identity of adult hybrids was also demonstrated by Wang et al. using SSR and ITS analyses [19]. In addition, cytoplasmic inheritance has been investigated using 16S rDNA, and the mode of mtDNA transmission has been shown to be matrilineal in scallops. Differing from scallops, mussels display two types of mtDNA genomes that are transmitted by males and females separately in a mode of transmission termed doubly uniparental inheritance [20], [21]. Taken together, the F1 hybrids from *A. purpuratus* × *A. i. irradians* inherited nuclear DNA from their parents and cytoplasmic genetic material maternally.

In our study, GISH demonstrated that both *A. purpuratus* and *A. i. irradians* had the same chromosome number and similar chromosome morphology, and their karyotypes were 2 n = 32 = 10 st+22 t (st represents subtelocentric chromosome, t represents telocentric chromosome), which were consistent with those previously reported [17], [18]. GISH also indicated that most *A. purpuratus* × *A. i. irradians* hybrids received one haploid genome from each parent and possessed 32 chromosomes; thus, the hybrids could be regarded as heterologous diploids. In studies of the interspecific hybridization of *M. nobilis* × *C. farreri* [12] and *P. yessoensis* × *C. farreri* [22], the number or morphology of the chromosomes was distinctly different in the two related species: *C. farreri* possessed 38 chromosomes, with a karyotype of 2 n = 38 = 6 m+10 sm+22 st (m represents metacentric chromosome, sm represents submetacentric chromosome) [23], and *M. nobilis* possessed 32 chromosomes, with a karyotype of 2 n = 32 = 6 m+26 t [24]. Moreover, *P. yessoensis* contained 38 chromosomes, but its karyotype was 2 n = 38 = 6 m+10 sm+16 st+6 t [10], [22]. Although the analysis of the genomic constitution of their hybrid larvae at early developmental stage indicated that these embryos received one haploid genome from each parent [10], [12], the combination of two divergent genomes could result in genomic instability [25], and the interaction of heterologous chromatin might be an important factor that leads to karyotype polymorphism in hybrid offspring [22]. In our study, some aneuploids and allopolyploids were observed in F1 hybrids, and the frequency of them was lower than that of the abnormal chromosomes detected in the hybrids of *M. nobilis* × *C. farreri* [12]. Chromosomal aneuploidy was found to be one of the most significant factors that might have a large effect on the embryo survival rate [26]–[28]. All these findings provide possible explanations for why the hybrid adults of *M. nobilis* × *C. farreri* and *P. yessoensis* × *C. farreri* were not readily obtained. Compared to these species, *A. purpuratus* and *A. i. irradians* have similar genomes and karyotypes, and they both belong to the *Argopecten* genus, implying that the nuclear genomes of these two scallops and the cytoplasmic genetic material from the maternal parent may have strong compatibility in the hybrid zygotes, resulting in viable hybrid adults.

In general, distant hybridization could result in genomic changes that include alterations in gene expression, chromosomal structure and genome size [29]–[32]. Sequence analysis of the ITS region in the *A. purpuratus* × *A. i. irradians* hybrids not only confirmed the presence of parental ITS sequences in the hybrid adults but also revealed some recombinant variants (Fig. 1). These recombinant variants were most likely intermediates of the paternal ITS region undergoing gene conversion, thus indicating that some alterations occurred in the hybrid genome. In fact, recombination intermediates have been reported for the ITS sequences of *C. farreri* × *A. irradians* hybrids [33]. Although gene conversion could be bidirectional, as reported in *Gossypium* allopolyploid and *Nicotiana* allopolyploid [34], [35], it was found maternally biased in the hybrids of *C. farreri* ♀ × *A. irradians ♂*; indeed, biased gene conversion is considered to be a general phenomenon in Pectinidae [33]. However, biased gene conversion was not found in our study because the maternal and paternal alleles were both present in the hybrid genomes and were nearly equal.

The occurrence of ITS recombinant variants in the hybrids was found through ITS sequencing, but was not detected by GISH in the present study. Eickbush and Eickbush [36] reviewed that unequal crossover has been regarded as the major mechanism for the evolution of rRNA genes. Wang et al. [33] proposed that the possible mechanism for biased gene conversion in the hybrid of *C. farreri* ♀ × *A. irradians ♂* might involve maternal restriction systems. In the initial formation of the hybrid, the maternal enzymatic system may treat the chromosomes of *A. irradians* as intruding chromosomes. Specific sites in paternal recombinant regions are thus recognized by maternal endonucleases to produce double-strand breaks. Biased gene conversion can subsequently occur when the directionality of double-strand break repair is in favor of the maternal DNA sequences. In the hybrids of *A. purpuratus* × *A. i. irradians*, further investigation needs to be conducted to obtain the real mechanism of the occurrence of ITS recombinant variants. In addition, as opposed to the biased gene conversion in the hybrid of *C. farreri* ♀ × *A. irradians ♂*, recombinant variants in the hybrids in our present study suggested that the two parental chromosomes might be compatible, with no dominant bias in either of them. Moreover, the appearance of recombinant variants in the hybrids might provide an explanation for the origin of hybrid zone novel alleles. The AFLP analysis of the hybrid genome in our other study also revealed the occurrence of alterations, mainly involved the loss of parental AFLP loci and the gain of novel loci (unpublished). These genetic variations might provide a possible basis for the heterosis observed in the *A. purpuratus* × *A. i. irradians* hybrids.

In the GISH analysis, it was found that the fluorescence signals were not uniform among the chromosomes, with stronger signals showing banding patterns on different chromosomes (Fig. 3). In fact, such phenomena of GISH banding have been observed in previous GISH studies, including the chromosomes of *Alstroemeria* [37], *Dasypyrum* [38] and rye [39] and in the F1 hybrids of *C. farreri* × *M. nobilis* [12]. GISH banding patterns were first reported by Kuipers et al. [37] that coincided with Giemsa C-banding patterns in the genus *Alstroemeria* using GISH with blocking DNA. Considering that GISH is a method primarily based on differences in repetitive sequences, stronger fluorescence signals might be initiated by uneven repetitive sequences clustering along the chromosome arms or on different chromosomes in the same cell. In our GISH experiment, stronger hybridization signals were found mainly in the telomeric and/or peri-telomeric regions of the *A. i. irradians* chromosomes and in the peri-centromeric and/or centromeric regions of the *A. purpuratus* chromosomes. These regions were not effectively blocked by blocking DNA, reflecting a more rapid rate of evolution and divergence in these heterochromatin regions [40] and species-specific or genome-specific repetitive sequences rapidly accumulating in closely related species [41], [42]. Huang et al. [12] found that the highly conserved heterochromatin sequences clustered in these regions may have produced uniform GISH signals across the chromosomes, as supported by the evidence of a sequencing study of a *C. farreri* fosmid library [43] in which species-specific DNA satellites were found and mapped to the chromosomes of *C. farreri* via FISH [44]. Based on the above data, we speculate that the strong GISH signals indicated heterochromatin regions across the chromosomes of *A. i. irradians* and *A. purpuratus* in which highly conserved species-specific sequences were clustered. Furthermore, the different GISH banding patterns in *A. i. irradians* and *A. purpuratus* suggested the different distribution of species-specific repeat sequences in the genomes of these two scallop species.

In conclusion, our study demonstrated that the hybrids between *A. purpuratus* and *A. i. irradians* were true hybrids at the cellular and molecular levels. The hybrid larvae possessed the expected chromosome constituents, and the hybrid adults inherited nuclear genetic material from both parents. Cytoplasmic inheritance in scallops was found to be maternal based on the analysis of mitochondrial 16S rDNA in the hybrids. Moreover, ITS sequence analysis indicated that some variations occurred in the hybrid genomes. This study represents the first step in analyzing the genetic basis of heterosis in these scallop hybrids. All these data could provide the theoretical foundation for further work, such as analyzing the genomic composition and genetic variation in the hybrids using the new generation of high-throughput sequencing technology, identifying the possible markers associated with heterosis and studying the coordination mechanism of hybrid genome during the combination of the two divergent genomes.

## Materials and Methods

### Scallop Materials

Several mature individuals of *A. purpuratus* (PP) and *A. i. irradians* (BB) were collected from the Jiaonan scallop hatchery in Qingdao, Shandong Province, China. *A. purpuratus* and *A. i. irradians* were induced to spawn, and insemination was performed according to the method outlined by Wang et al. [19]. After the collection of gametes, the eggs from one species were fertilized by adding sperm from the other species to produce hybrids (*A. purpuratus* ♀ × *A. i. irradians* ♂, PB and *A. i. irradians* ♀ × *A. purpuratus* ♂, BP) or those from the same species to produce inbred cohorts. The resulting larvae were reared following routine culture procedures, as described by Zhang et al. [45]. A portion of the embryos was collected at the trochophore stage for chromosomal preparations, and adult hybrid scallops were obtained after 6 months.

### DNA Extraction

Only adult specimens were used for DNA preparation. The adductor muscles were removed from the living individuals and stored in liquid nitrogen until use. DNA was extracted according to the traditional phenol/chloroform extraction method [46].

### PCR Amplification, Cloning and Sequencing

The ITS region and a fragment of 16S rDNA were amplified for forty individuals, including PP, BB, PB and BP (10 individuals each group). The ITS primers were those of Wang et al. [33] (forward, 5′-GTTTCTGTAGGTGAACCTG-3′; reverse, 5′-CTCGTCTGATCTGAGGTCGGA-3′), and the amplification of 16S rDNA used the universal primers 5′-CGCCTGTTTATCAAAAACAT-3′ and 5′-CCGGTCTGAACTCAGATCACGT-3′ [47]. The PCR amplifications were performed in a 50 µl volume with 100 ng DNA template, 1.5 mM MgCl_2_, 0.4 µM each primer, 0.2 mM each dNTP, 1.0 U Taq DNA polymerase (TaKaRa, Dalian, China) and 1× universal PCR buffer. The PCR program was 5 min at 94°C followed by 30 cycles of 40 s at 94°C, 40 s at the annealing temperature and 60 s at 72°C, with a final extension of 10 min at 72°C. The annealing temperatures were 54°C and 55°C for the amplifications of the ITS region and 16S rDNA, respectively. The amplified fragments were evaluated by electrophoresis through 1% agarose gels and visualized using ethidium bromide. Additionally, a portion of the amplified ITS fragments was separated on a 10% non-denaturing polyacrylamide gel at 200 V for 2 h, stained with ethidium bromide and visualized under ultraviolet light. The sizes of the bands were determined with the software QUANTITY ONE (Bio-Rad). The ITS and 16S rDNA PCR products were then ligated into pMD18-T (TaKaRa, Dalian, China) and transferred into competent *Escherichia coli* DH5α. Positive colonies were sequenced using a 3730 automatic sequencer (ABI, USA). Sequence alignments were performed using the program ClustalX 1.81 [48].

For the ITS analysis, the PCR products of all PP and BB individuals and six random hybrid individuals (3 PB and 3 BP) were cloned after transformation, and three independent clones from each PP and BB individual and sixteen independent clones from each PB and BP individual were randomly selected for sequencing. For the 16S rDNA analysis, all tested individuals were cloned and sequenced. The molecular diversity indices, such as polymorphic sites, transitions, transversions and indels, and the genetic distance (Kimura 2-parameter) among these four groups were analyzed using MEGA 3 [49]. The phylogenetic tree was constructed using MrBayes version 3.2.1 [50].

### Chromosome Preparations and GISH

The progeny individuals were sampled at the trochophore stage and used for GISH analysis. Briefly, the larvae were treated with colchicine (0.01%) at room temperature for 2 h and KCl (0.075 M) for 25 min, and then fixed three times (15 min each) in Carnoy’s fixative (methanol: glacial acetic acid = 3∶1 v/v). The fixed larvae were dissociated in 50% acetic acid, and the dissociated cell suspension was dropped onto hot wet glass slides and air-dried.

The total genomic DNA of *A. i. irradians* and *A. purpuratus* was labeled with digoxigenin-11-dUTP and biotin-16-dUTP using the nick translation kits (Roche) following the manufacturer’s instructions. The length of the probes for GISH was between 100 bp and 600 bp, which were estimated by 2% agarose gel electrophoresis. The labeled probes were resolved at a concentration of 5 ng/ml in a hybridization solution of 2 × SSC, 50% deionized formamide, 10% dextran sulfate and 500 ng/ml salmon testis DNA (Amresco). Unlabeled blocking DNA was generated by sonicating DNA into approximately 100–200 bp fragments, which were added to the probe solution. GISH was performed according to the protocol described by Bi and Bogart [51]. The fluorescent signals were detected with anti-digoxigenin rhodamine (Roche) or avidin-FITC (Vector), and the chromosomes were counterstained with DAPI or PI (Vector). The hybridization signals were observed using a Nikon E-600 epi-fluorescence microscope equipped with the appropriate filters. The digital images were captured using a CCD camera (COHU) and analyzed with Lucia FISH software. More than 50 spreads were examined for each sample.

## Supporting Information

Figure S1 **Electrophoreses of amplified ITS products from** ***A. purpuratus*** **,** ***A. i. irradians*** **and their hybrids.** M1, DL2000 marker; M2, 100 bp DNA ladder; lanes 1–4, *A. purpuratus*; lanes 5–8, *A. i. irradians*; lanes 9–12, *A. purpuratus* ♀ × *A. i. irradians* ♂; lanes 13–16, *A. i. irradians* ♀ × *A. purpuratus* ♂.
(TIF)

## References

[1] Bartley DM, Rana K, Immink AJ (2001) Interspecific hybrids in aquaculture and fisheries. Reviews in Fish Biology and Fisheries 10: 325–337.

[2] Epifanio J, Nielsen J (2000) The role of hybridization in the distribution, conservation and management of aquatic species. Reviews in Fish Biology and Fisheries 10: 245–251.

[3] Sittikraiwong P (1987) Karyotype of the hybrid between *Clarias macrocephalus* gunther and *Pangasius sutchi* Fowler: [M. S. thesis]. Bangkok, Thailand, Kasetsart University.

[4] Stanley JG (1976) Production of hybrid, androgenetic, and gynogenetic grass carp and carp. Trans Am Fish Sac 105(1): 10–16.

[5] Ye Y, Wu Q, Chen R (1989) Studies on cytology of crosses between grass carp and carp-asynchronization between nucleus and cytoplasm in distant hybridization of fishes. Acta Hydrobiologica Sinica 13(3): 234–239.

[6] Xu D, You F, Wu Z, Li J, Ni J, et al (2009) Genetic characterization of asymmetric reciprocal hybridization between the ﬂatfishes *Paralichthys olivaceus* and *Paralichthys dentatus* . Genetica137: 151–158. 10.1007/s10709-009-9373-z 19488828

[7] Bi K, Bao Z, Huang X, Wang J, Zhao Y, et al (2005) Cytological observation on cross fertilization and the development of early embryos of *Chlamys farreri* ♀ × *C. nobilis* ♂ with fluorescent microscope. J Ocean Univ Qingdao 35: 283–286.

[8] Wang J, Bi K, Huang X, Zhang L, Hu J, et al (2006) Cytological observation on hybrid fertilization *Chlamys nobilis* ♀ × *C. farreri* ♂ with fluorescent microscope. Biotech Bull 6: 122–126.

[9] Lü Z, Yang A, Wang Q, Liu Z, Zhou L (2006a) Preliminary cytological identification and immunological traits of hybrid scallop from *Chlamys farreri* (♀)× *Patinopecten yessoens* (♂). Journal of Fishery Sciences of China 13(4).

[10] Lü Z, Yang A, Wang Q, Liu Z, Zhou L (2006b) Cytogenetic analysis on the hybrids from reciprocal crosses of *Chlamys farreri* × *Patinopecten yessoens* . High Tech Lett 16(8): 853–858.

[11] Liu X, Chang Y (2006) The feasibility of the hybridization in four species of scallop and the early development of their offsprings. Journal of Dalian Fisheries University 21(4): 346–349.

[12] Huang X, Bi K, Hu L, Sun Y, Lu W, et al (2011) Fertilization and cytogenetic examination of interspecific reciprocal hybridization between the scallops, *Chlamys farreri* and *Mimachlamys nobilis.* . PLoS ONE 6(11): e27235. 22110617 10.1371/journal.pone.0027235 PMC3215693

[13] Yang A, Wang Q, Liu Z, Zhou L (2004) The hybrid between the scallops *Chlamys farreri* and *Patinopecten yessoensis* and the inheritance characteristics of its first filial generation. Mar Fish Res 25: 1–5.

[14] Waller TR (1969) The evolution of the *Argopecten gibbus* stock (Mollusca: Bivalvia), with emphasis on the Tertiary and Quaternary species of eastern North America. J Paleontol 43 Suppl. to No. 5, Mem. 3: 1–125.

[15] Zhang F, He Y, Liu X, Ma J, Li S, et al (1986) A report on the introduction, seed culture and experimental grow-out of the bay scallop, *Argopecten irradiands* Larmarck. Oceanol Limnol Sin 17: 367–374.

[16] Dall WH (1909) The mollusca and branchiopoda. Report of the dredging operation, ‘Albatros’ 1891. Bull Mollusca Comp Zool 37: 147–294.

[17] Gajardo G, Parraguez M, Colihueque N (2002) Karyotype analysis and chromosome banding of the Chilean-Peruvian scallop *Argopecten purpuratus* (Lamarck, 1819). J Shellfish Res 21: 585–590.

[18] Wang Y, Guo X (2004) Chromosomal rearrangement in Pectinidae revealed by rRNA loci and implications for bivalve evolution. Biol Bull 207: 247–256. 15616355 10.2307/1543213

[19] Wang C, Liu B, Li J, Liu S, Li J, et al. (2011) Introduction of the Peruvian scallop and its hybridization with the bay scallop in China. Aquaculture doi: 310: 380–387.

[20] Skibinski DOF, Gallagher C, Beynon CM (1994) Sex-limited mitochondrial DNA transmission in the marine mussel Mytilus edulis. Genetics 138: 801–809. 7851776 10.1093/genetics/138.3.801 PMC1206229

[21] Zouros E, Ball AO, Saavedra C, Freemann KR (1994) An unusual type of mitochondrial DNA inheritance in the blue mussel Mytilus. Proc Natl Acad Sci USA 91: 7463–7467. 8052604 10.1073/pnas.91.16.7463 PMC44421

[22] Zhao Y, Bao Z, Bi K, Huang X, Wang J, et al (2006) Karyotypes of hybid scallop (hybridizing cross the female *Patinopecten yessoensis* with the male *Chlamys farreri*) and their parents. Acta Oceanol Sin 28(1): 100–105.

[23] Wang M, Zheng J, Yu H (1990) The karyotype of Chlamys farreri. J Ocean Univ Qingdao 20: 81–85.

[24] Komaru A, Wada KT (1985) Karyotypes of four species in the Pectinidae (Bivalvia: Pteriomorphia). Venus Jap J Malac 44: 249–259.

[25] Marfil CF, Masuelli RW, Davison J, Comai L (2006) Genomic instability in *Solanum tuberosum* × *Solanum kurtzianum* interspecific hybrids. Genome 49: 104–113. 16498460 10.1139/g05-088

[26] Leitao A, Boudry P, Thiriot-Quie ´vreux C (2001) Negative correlation between aneuploidy and growth in the Pacific oyster, *Crassostrea gigas*: ten years of evidence. Aquaculture 193: 39–48.

[27] Gardner RJM, Sutherland GR (2004) Chromosome abnormalities and genetic counseling. Oxford: Oxford University Press. 596 p.

[28] McCombie H, Lape `gue S, Cornette F, Ledu C, Boudry P (2005) Chromosome loss in bi-parental progenies of tetraploid Pacific oyster *Crassostrea gigas* . Aquaculture 247: 97–105.

[29] Fujiwara A, Abe S, Yamaha E, Yamazaki F, Yoshida MC (1997) Uniparental chromosome elimination in the early embryogenesis of the inviable salmonid hybrids between masu salmon female and rainbow trout male. Chromosoma 106: 44–52. 9169586 10.1007/s004120050223

[30] Rieseberg LH (2001) Chromosomal rearrangements and speciation. Trends Ecol Evol 16: 351–358. 11403867 10.1016/s0169-5347(01)02187-5

[31] Baack EJ, Rieseberg LH (2007) A genomic view of introgression and hybrid speciation. Curr Opin Genet Dev 17: 513–518. 17933508 10.1016/j.gde.2007.09.001 PMC2173880

[32] Chen L, Lou Q, Zhuang Y, Chen J, Zhang X, et al (2007) Cytological diploidization and rapid genome changes of the newly synthesized allotetraploids *Cucumis* × *hytivus* . Planta 225: 603–614. 16953430 10.1007/s00425-006-0381-2

[33] Wang S, Zhang L, Hu J, Bao Z, Liu Z (2010) Molecular and cellular evidence for biased mitotic gene conversion in hybrid scallop. BMC Evolutionary Biology 10: 6. 20064268 10.1186/1471-2148-10-6 PMC2818637

[34] Wendel JF, Schnabel A, Seelanan T (1995) Bidirectional interlocus concerted evolution following allopolyploid speciation in cotton (Gossypium). Proc Natl Acad Sci USA 92(1): 280–284. 7816833 10.1073/pnas.92.1.280 PMC42862

[35] Kovarik A, Matyasek R, Lim KY, Skalick K, Koukalov B, et al (2004) Concerted evolution of 18–5.8–26S rDNA repeats in *Nicotiana* allotetraploids. Biological Journal of the Linnean Society 82: 615–625.

[36] Eickbush TH, Eickbush DG (2007) Finely orchestrated movements: evolution of the ribosomal RNA genes. Genetics 175: 477–85. 17322354 10.1534/genetics.107.071399 PMC1800602

[37] Kuipers AGJ, van Os DPM, de Jong JH, Ramanna MS (1997) Molecular cytogenetics of Alstroemeria: identification of parental genomes in interspecific hybrids and characterization of repetitive DNA families in constitutive heterochromatin. Chromosome Res 5: 31–39. 9088641 10.1023/a:1018489318300

[38] Minelli S, Ceccarelli M, Mariani M, De Pace C, Cionini PG (2005) Cytogenetics of *Triticum* × *Dasypyrum* hybrids and derived lines. Cytogenet Genome Res 109: 385–392. 15753601 10.1159/000082424

[39] Zhou J, Yang Z, Li G, Liu C, Ren Z (2008) Discrimination of Repetitive Sequences Polymorphism in *Secale cereale* by Genomic In Situ Hybridization-Banding. Journal of Integrative Plant Biology 50(4): 452–456. 18713379 10.1111/j.1744-7909.2008.00644.x

[40] Ferree PM, Barbash DA (2009) Species-specific heterochromatin prevents mitotic chromosome segregation to cause hybrid lethality in *Drosophila* . PLoS Biol 7(10): e1000234. 19859525 10.1371/journal.pbio.1000234 PMC2760206

[41] Ragghianti M, Guerrini F, Bucci S, Mancino G, Hotz H, et al (1995) Molecular characterization of a centromeric satellite DNA in the hemiclonal hybrid frog *Rana esculenta* and its parental species. Chromosome Res 3: 497–506. 8581303 10.1007/BF00713965

[42] Targino VG, Henrique SC, Claudia GM, Feldberg E, Martins C (2009) Comparative cytogenetics of cichlid fishes through genomic in situ hybridization (GISH) with emphasis on *Oreochromis niloticus* . Chromosome Res 17: 791–799. 19685270 10.1007/s10577-009-9067-5

[43] Zhang L, Chen C, Cheng J, Wang S, Hu X, et al (2008a) Initial analysis of tandemly repetitive sequences in the genome of Zhikong scallop (*Chlamys farreri* Jones et Preston). DNA Seq 19: 195–205. 17852361 10.1080/10425170701462316

[44] Zhang L, Bao Z, Wang S, Hu X, Hu J (2008b) FISH Mapping and identification of Zhikong Scallop (*Chlamys farreri*) chromosomes. Mar Biotechnol 10: 151–157. 17955291 10.1007/s10126-007-9045-x

[45] Zhang H, Liu X, Zhang G, Wang C (2007) Growth and survival of reciprocal crosses between two bay scallops, *Argopecten irradians concentricus* Say and *Argopecten irradians irradians* Lamarck (1819). Aquaculture 272: S88–S93.

[46] Sambrook J, Fritsch EF, Maniatis T (1989) Molecular Cloning: A Laboratory Manual 2^nd^ edn. Cold Springs Harbour, New York: Cold Springs Harbour Laboratory Press.

[47] Palumbi SR, Martin A, Romano S (1991) The Simple Fool’s Guide to PCR. Honolulu, HI: University of Hawaii Press.

[48] Thompson JD, Gibson TJ, Plewniak F, Jeanmougin F, Higgins DG (1997) The ClustalX windows interface: flexible strategies for multiple sequence alignment aided by quality analysis tools. Nucleic Acids Research 24: 4876–4882. 10.1093/nar/25.24.4876 PMC147148 9396791

[49] Kumar S, Tamura K, Nei M (2004) MEGA3: integrated software for molecular evolutionary genetics analysis and sequence alignment. Brief Bioinform 5: 150–163. 15260895 10.1093/bib/5.2.150

[50] Ronquist F, Teslenko M, van der Mark P, Ayres DL, Darling A, et al (2012) MrBayes 3.2: efficient Bayesian phylogenetic inference and model choice across a large model space. Syst Biol 61 (3): 539–542. 10.1093/sysbio/sys029 PMC3329765 22357727

[51] Bi K, Bogart JP (2006) Identification of intergenomic recombinations in unisexual salamanders of the genus *Ambystoma* by genomic in situ hybridization (GISH). Cytogenet Genome Res 112: 307–312. 16484787 10.1159/000089885

