# Supplementary figures and images for: Genomic Characterization of Interspecific Hybrids between the Scallops Argopecten purpuratus and A. irradians irradians
Source: PLoS One. 2013 Apr 19;8(4):e62432. doi: 10.1371/journal.pone.0062432 (PMC3631176; doi:10.1371/journal.pone.0062432)

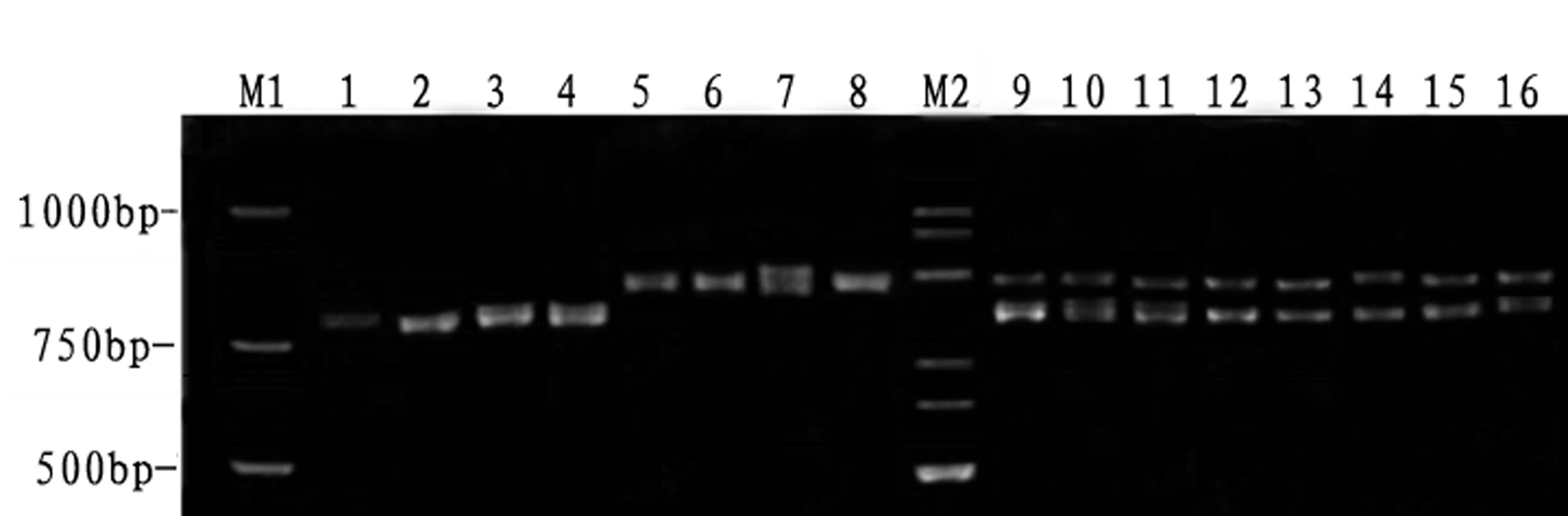

Supplement: Figure S1 — Electrophoreses of amplified ITS products from A. purpuratus , A. i. irradians and their hybrids. M1, DL2000 marker; M2, 100 bp DNA ladder; lanes 1–4, A. purpuratus; lanes 5–8, A. i. irradians; lanes 9–12, A. purpuratus ♀ × A. i. irradians ♂; lanes 13–16, A. i. irradians ♀ × A. purpuratus ♂. (TIF) [file pone.0062432.s001.tif]
